# A cross-sectional study to estimate high-risk human papillomavirus prevalence and type distribution in Italian women aged 18–26 years

**DOI:** 10.1186/1471-2334-13-74

**Published:** 2013-02-07

**Authors:** Cristina Giambi, Serena Donati, Francesca Carozzi, Stefania Salmaso, Silvia Declich, Marta L Ciofi degli Atti, Guglielmo Ronco, Maria P Alibrandi, Silvia Brezzi, Natalina Collina, Daniela Franchi, Amedeo Lattanzi, Maria C Minna, Roberto Nannini, Elena Barretta, Elena Burroni, Anna Gillio-Tos, Vincenzo Macallini, Paola Pierotti, Antonino Bella

**Affiliations:** 1 Communicable Disease Epidemiology Unit, National Centre for Epidemiology, Surveillance and Health Promotion; Istituto Superiore di Sanità, Viale Regina Elena 299, 00161 Rome, Italy; 2 Analytical Cytology and Biomolecular Unit, Cancer Research and Prevention Institute, Via Cosimo il Vecchio 2, 50139 Florence, Italy; 3 Medical Direction, Bambino Gesù Children’s Hospital, Piazza S. Onofrio 4, 00165 Rome, Italy; 4 Unit of cancer epidemiology, Center for Cancer Prevention, S. Francesco da Paola 31, 10123 Turin, Italy; 5 Hygiene and Public Health Unit, LHU Torino4, Via Aldisio 2, Ivrea, 10015 Turin, Italy; 6 Department of Prevention, LHU Viterbo, via Enrico Fermi 15, 01100 Viterbo, Italy; 7 Public Health Department, LHU Bologna, Via del Seminario 1, San Lazzaro di Savena, 40068, Bologna, Italy; 8 Department of Prevention, LHU Avezzano-Sulmona-L’Aquila, Via Monte Velino 18, 67051 Avezzano, L’Aquila, Italy; 9 San Liberatore Hospital, LHU Teramo, Viale Risorgimento, 64032 Atri, Teramo, Italy; 10 Community Medicine Unit, LHU Pescara, Via Paolini 45, 65124 Pescara, Italy; 11 Oncologic Screening Center, LHU Imola, viale Amendola 8, 40026 Imola, Bologna, Italy; 12 Mother and Child Department, LHU Napoli 2 Nord, Corso Italia 129, 80010 Quarto, Napoli, Italy; 13 Center for Experimental Research and Medical Science, University of Turin, Via Santena 7, 10126 Turin, Italy; 14 S. Rinaldi Hospital, Via Serafino Rinaldi 63, 67057 Pescina, L’Aquila, Italy; 15 Maggiore Hospital, LHU Bologna, Largo B. Nigrisoli 2, 40133 Bologna, Italy

**Keywords:** Human papillomavirus, High-risk types, Prevalence, Genotype distribution, Cervical cancer, Italy, Risk factors

## Abstract

**Background:**

Pre-vaccination information on HPV type-specific prevalence in target populations is essential for designing and monitoring immunization strategies for cervical cancer (CC) prevention. Data on HPV prevalence in Italy are available for women over the age of 24 years, target of the population-based CC screening programmes; while data of HPV prevalence in younger ages are very limited. The present study enrolled Italian women aged 18–26 years in order to assess the prevalence and distribution of high-risk (HR) HPV types. Risk-factors correlated with HR-HPV positivity were also described.

**Methods:**

A sample of 2,289 women was randomly selected from the resident population lists of ten Local Health Units (LHUs) located in six Italian Regions scattered across the country; both rural and urban LHUs were involved. Women aged between 18 and 26 years and living in the selected LHUs were included in the study; pregnant women and women who did not speak Italian were excluded. A total of 1,102 women met the inclusion criteria and agreed to participate. Participants were offered pap test and Hybrid-Capture 2 (HC2) test for HR-HPV types and genotyping was performed on positive smears.

**Results:**

Out of 1,094 valid samples, 205 (18.7%) were HR-HPV positive. Women with 2–4 (OR_adj_ = 4.15, 95%CI: 2.56-6.72) and ≥5 lifetime partners (OR_adj_ = 10.63, 95%CI: 6.16-18.36) and women who have used any contraceptive in the last six months (OR_adj_ = 1.67, 95%CI: 1.09-2.54) had a higher risk to be infected; women living with their partner had a lower risk (OR_adj_ = 0.56, 95%CI: 0.34-0.92) to acquire infection than women living with parents/friends/alone. Among HC2 positive women, HPV16 was the most prevalent type (30.9%), followed by 31 (19.6%), 66 (12.9%), 51 (11.3%), 18 (8.8%), 56 (8.8%). Co-infections of HR-HC2 targeted types were found in 20.4% of positive samples. The HR-HPV prevalence in women with abnormal cytology (52.4%) was significantly higher than in women with normal cytology (14.6%); however 33.0% of HR-HPV infected women had an abnormal cytology.

**Conclusion:**

HR-HPV prevalence in Italian women aged 18–26 years was 19%, higher than what detected for older women, by other studies using the same molecular method and laboratory network; this result supports the choice of electing girls before the sexual debut as the primary target of HPV vaccination. The HPV type distribution found in this study may represent a baseline picture; an accurate post-vaccine surveillance is necessary to early detect a possible genotype replacement. The high prevalence of viral types other than vaccine-HPV types supports the necessity to guarantee the progression of CC screening programmes in vaccinated women.

## Background

In the last decade many studies have shown that infection with high-risk (HR) types of human papillomavirus (HPV) is a necessary condition for the development of invasive cervical cancer (CC) [1]. Genital HPV infection is very common in sexually active women and is transient in most cases; only women with persistent infection by HR-HPV types are likely to develop a cervical lesion [2]. These findings led to the development of prophylactic vaccines targeted to provide protection against HPV16 and 18, responsible for the 70% of all cervical cancers; efficacy has been shown to be highest in subjects naïf to the infection [3,4].

As of July 2010, 18 European countries had integrated HPV vaccination in their national immunization programmes, most of them targeting pre-adolescent girls; nine countries had also planned catch up programmes for older girls [5]. In Italy HPV vaccination is included in the national immunization program and is offered to 11 year-old girls since 2007 [6]; six of the 21 Italian Regions have also extended the offer to one older birth cohort (varying by Region between 15–18 years), and one Region to three cohorts (15, 18 and 24 year-old) [7].

Pre-vaccination distribution of HPV genotypes in target populations is essential for designing, monitoring and evaluating immunization strategies. Baseline prevalence is relevant to estimate vaccine effectiveness against the HPV-vaccine types, to evaluate the cross-protection of the vaccines, and to monitor over time the relative frequency of genotypes under the selective pressure of the vaccines.

Data on HPV prevalence in Italy are available for women over the age of 24 years [8-10], target of the population-based CC screening programmes. On the contrary there is very limited amount of data on HPV prevalence in younger ages [11,12]. The present study enrolled Italian women aged 18–26 years in order to assess the prevalence of HR-HPV types and detect the prevalent genotypes. Risk-factors correlated with HR-HPV positivity were also described.

## Methods

### Study design and study population

This cross-sectional study was carried out in the period 2007–2009, coordinated by the Istituto Superiore di Sanità (ISS, Italy’s National Institute of Public Health) and partially sponsored by the Ministry of Health. It was part of a national project (PreGio), which also included a KAP (knowledge-attitudes-practices) survey on HPV infection and CC prevention [13] and a study to estimate the acceptance rate of HPV vaccination [14] .

A sample of 2,289 women was randomly selected from the resident population lists of ten Local Health Units (LHU) located in six Italian Regions scattered across the country: Abruzzo and Campania in southern Italy (1,026), Lazio and Tuscany in central Italy (388) and Emilia-Romagna and Piedmont in northern Italy (875). Both rural and urban LHUs were involved; all 10 LHUs have population-based CC screening programmes.

The inclusion criteria for the study were: women aged between 18 and 26 years and living in the selected LHUs. Pregnant women and women who did not speak Italian were excluded. The women were stratified into two age groups (18–24 and 25–26 years), partially overlapping (25–26 years) with the target age of population-based CC screening. Procedures for enrolling participants as well as study methodology have already been described [13,14].

All women were offered Pap-test and HPV test; a written informed consent was obtained by each participant. Selected midwives, especially trained for this study, were in charge of contacting sampled women, collecting socio-demographic data and information regarding sexual and reproductive history, getting informed consent and performing cervical smears. The study was approved by the national ethics committee of the ISS.

### Cytology

Cervical smears were taken according to the consolidate CC-screening procedures at the LHU. In most Regions (Tuscany, Piedmont, Emilia-Romagna, Lazio, Campania) two cervical samples were taken: one for Pap-test and one for the HPV test in Specimen Transport Medium (STM, DNAPAP cervical sampler, Qiagen, Gaithersburg, USA); for STM sample, cytobrush was used to obtain samples. In Abruzzo Region the cervical cell specimens were put in PreservCyt solution (Hologic, Inc., Marlborough, MA) and used both for HPV testing and for cytological examination; for Preservcyt sample, Ayre spatula and cytobrush were used. The cytology was interpreted locally according to Bethesda 2001 system [15]. Information on reproductive, sexual and cervical screening history were collected (Table 1).

**Table 1:** Characteristics of the study population

**Characteristics**		**n (%)**	**N**
**Socio-demographic characteristics**			
Mean age (years)		23.8 ± 0.07 *	1,094
Age group	18–24 years	598 (54.7)	1,094
	25–26 years	496 (45.3)	
Geographical area of residence	North	445 (40.7)	1,094
	Centre	235 (21.5)	
	South	414 (37.8)	
Nationality	Foreign	69 (6.3)	1,092
	Italian	1,023 (93.7)	
Educational level	Low (≤8 years)	216 (19.8)	1,090
	High (>8 years)	874 (80.2)	
Employment status	Student	455 (42.0)	1,084
	Employed	415 (38.3)	
	Unemployed	163 (15.0)	
	Housewife	51 (4.7)	
Marital status	Single	974 (89.3)	1,091
	Married	113 (10.3)	
	Divorced	4 (0.4)	
Lives with	parents	813 (74.7)	1,089
	partner	185 (17.0)	
	alone	48 (4.4)	
	friends	43 (3.9)	
**Sexual behaviour and reproductive history**			
Mean age at first sexual intercourse (years)		17.5 ± 0.1 *	1,089
Parity	0	988 (90.4)	1,093
	≥1child	105 (9.6)	
Mean age at first delivery (years)		21.7 ± 0.3 *	105
No. of lifetime partners	1	377 (34.6)	1,091
	2–4	558 (51.1)	
	≥5	156 (14.3)	
No. of partners in the last 6 months	0	69 (6.3)	1,093
	1	962 (88.0)	
	2–4	53 (4.9)	
	≥5	9 (0.8)	
Use of contraceptives in the last 6 months	Yes	762 (74.5)	1,023
	No	261 (25.5)	
Use of condoms in the last 6 months	Always	346 (33.9)	1,020
	Rarely	307 (30.1)	
	Never	369 (36.0)	
Previous Pap-test	Yes	420 (38.5)	1,091
	No	671 (61.5)	

* (mean ± Standard Error).

Women aged 25–26 years followed the routine protocol of treatment or follow-up according to cytological results. A specific protocol for 18–24 year-old women was adopted: colposcopy was offered to women with HSIL (high squamous intraepithelial lesions) and ASCH (atypical squamous cells that cannot exclude high-grade lesions), while women with LSIL (low squamous intraepithelial lesions) or less severe lesions were invited to repeat pap-test two years later and colposcopy was offered only if, after two years, the diagnosis was still LSIL or it progressed to a more severe lesion.

### HPV testing

The HPV test was performed using Hybrid Capture 2 (HC2 High-Risk HPV DNA, Qiagen; Hilden, Germany) according to the manufacturer’s instructions. It is a hybridization assay which detects the presence of HPV-DNA using cocktails of RNA probes and an amplified, chemiluminescent signal. The high-risk group of probes B, designed to detect HPV types 16, 18, 31, 33, 35, 39, 45, 51, 52, 56, 58, 59 and 68, was used. The assay is calibrated on a positive cut off (pc) of 1 pg/ml of HPV-DNA. Samples were considered positive when the ratio between the Relative Light Units (RLU) of specimen and the pc attained or exceeded the value of 1.0.

A regional laboratory was identified in Tuscany, Piedmont, Abruzzo and Emilia-Romagna Regions for performing HPV testing; the Analytical and Biomolecular Cytology Unit of the Cancer Prevention and Research Institute (ISPO) in Tuscany also analysed samples from Campania and Lazio Regions.

### Genotyping

Genotyping was performed on HR-HC2 positive samples. DNA was extracted using the QIAamp DNAMini kit (Qiagen) according to the manufacturer’s instructions. For PreservCyt™ samples the elution volume varied from 80 to 100 μl depending on the size of the pellet, while for STM samples 100 μl of elution buffer (Buffer AE) were used. To facilitate a higher recovery of DNA a double elution was done for all the samples. HR-HC2 positive samples were typed using the “Consensus High Risk HPV genotyping” kit assay (Digene Corporation, Gaithersburg, USA). The test is based on the reverse hybridization principle. Denaturated biotynilated amplicons, resulting from the amplification of a part of the L1 region with GP5+/GP6+ primers, were hybridized with specific oligonucleotide probes, which were immobilized as parallel lines on membrane strips (Reverse Line Blot Hybridization). The hybrids were detected with alkaline phosphatase–streptavidin conjugate and substrate (5-bromo-4-chloro-3-indolylphosphate and nitroblue tetrazolium), resulting in a purple precipitate at positive probe lines. The kit allows the identification of 18 HPV types (16, 18, 26, 31, 33, 35, 39, 45, 51, 52, 53, 56, 58, 59, 66, 68, 73, 82). One HPV-positive control and two negative PCR controls (a purified DNA sample negative for HPV and a DNA free sample) were included in each PCR run and subsequent Reverse Line Blot (RLB). GP5+/GP6+ PCR-negative and RLB-negative samples were amplified for the β-globin gene sequence using GH20-PC04 primers (268-bp amplicon length) to assess DNA integrity (Bauer).

The molecular Laboratory of ISPO performed genotyping of samples collected in all Italian regions involved in the study except samples collected in Piedmont Region, which were genotyped at the Center for Experimental Research and Medical Science of the Turin University. ISPO also coordinated the molecular activities of the whole project, standardizing the procedures for storing and analysing the samples according to a protocol defined to guarantee the quality of molecular methods [16].

### Statistical analysis

Categorical variables were summarized by absolute frequencies and percentages, and continuous variables by means and Standard Error (SE). The Chi-square test and Fisher’s exact test were used to compare proportions; the *t*-test or Mann–Whitney nonparametric test was used to compare continuous variables. Crude and adjusted odds ratios (OR) and 95% confidence intervals (95%CI) were calculated to evaluate the association between the HR-HPV positivity and socio-behavioural characteristics.

Confounding was assessed by a multivariate logistic regression approach; all variables with a p-value ≤0.15 in the univariate analysis were included in the multivariate model and retained in the final model according to a log-likelihood-ratio test for goodness-of-fit. Statistical analysis was performed using STATA 11.2 (Stata Corporation, College Station, Texas, USA).

## Results

### Study population

**Figure 1:**
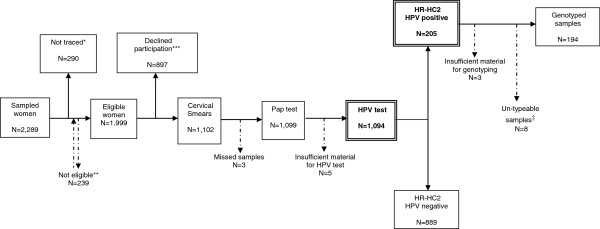
**Study population.** *Not traced: women who were not traced although three phone calls and two home visits. **Not eligible: women who did not meet inclusion criteria and were replaced with women of the same age group and local health unit. ***Declined participation: women who were contacted and met the inclusion criteria, but refused to participate in the prevalence study. ^‡^Un-typeable samples: HR-HC2 positive samples that resulted negative at any of the 18 types identifiable by the kit assay used for genotyping.

Recruitment was performed in the period ranging from February 2008 to April 2009. The initial sample included 2,289 women; 239 did not meet the inclusion criteria (124 did not live in the selected LHU, 57 were pregnant, 45 were not included in the 18–26 years range of age and 13 did not speak Italian) and were replaced, and 290 could not be contacted. Thus 1,999 women were offered Pap test and HPV test. Among them, 897 (44.9%) declined participation for several issues (non interested in the study, practical constraints, health disorders, HPV-positivity, recent previous Pap-test, embarrassment, parental advice, safety concerns, no sexual activity) (Figure 1). Women who did not participate were slightly younger than participants (mean age 23.2 ± 0.09 vs. 23.9 ± 0.07 years; p < 0.001); the proportion of women with a lower education level (24.6% vs 19.9%; p = 0.017), living with parents (87.9 vs 74.6%; p = 0 < 001), unmarried (93.5 vs 89.5%; p = 0.003) and living in southern Italy (53.3% vs 38.0%; p < 0.001) was higher in the group of women who refused to participate.

In conclusion a total of 1,102 out of 2,289 women (48.1%) were enrolled in the study. In order to calculate the participation rate to the prevalence study, we considered more appropriate to exclude from the denominator (2,289) the 154 women (among the 897 women who declined participation) who spontaneously declared not to be sexually active: therefore the participation rate was 51.6% (1,102/2,135), with a range among LHUs of 23.9-80.7.

The mean age of participants was 23.8 ± 0.07 years; 40.7% lived in northern Italy, 37.8% in southern Italy, and 21.5% in central Italy. Most women were Italian (93.7%), unmarried (89.3%) and had a high educational level, defined as > 8 years of education (80.2%). Detailed socio-behavioural characteristics are shown in Table 1.

### HPV prevalence and genotyping

The Pap-test was performed on 1,099 cervical smears because three samples were missed (they were included in the CC screening programme, instead of being included in the PreGio study) and the HR-HPV testing was performed on 1,094 out of 1,999 samples (the material of five samples was not sufficient for HR-HC2 test). The proportion of HR-HC2 positive samples was 18.7% (205/1,094) and did not differ by geographic area and age group (Table 2).

**Table 2:** Determinants of HR-HPV prevalence: univariate (OR crude) and multivariate (OR adj) logistic regression analysis

**Characteristics**		**HR-HPV positivity**	**Univariate analysis**		Multivariate model**	
		**n/N (%)**	**OR **_**crude**_	**95%CI**	**OR **_**adj**_	**95%CI**
Age Group	18-24 years	119/597 (19.9)	1	-		
	25-26 years	86/496 (17.3)	0.84	(0.62 – 1.14)		
Geographical area of residence	Centre	44/235 (18.8)	1	-		
	North	86/445 (19.3)	1.03	(0.69 – 1.55)		
	South	75/414 (18.1)	0.95	(0.63 – 1.44)		
Nationality	Foreign	13/69 (18.8)	1	-		
	Italian	192/1023 (18.7)	0.99	(0.53 – 1.86)		
Educational level	Low (≤8 years)	39/216 (18.1)	1	-		
	High (>8 years)	166/874 (19.0)	1.06	(0.72 – 1.56)		
Employment status	Student	79/455 (17.4)	1	-		
	Employed	80/415 (19.3)	1.14	(0.80 – 1.60)		
	Other	45/214 (21.0)	1.27	(0.84 – 1.91)		
Marital status	Single	197/974 (20.2)	1	-		
	Married	8/113 (7.1)	0.30	(0.14 – 0.63)		
Lives with	Parents/friends/alone	182/904 (20.1)	1	-	1	-
	Partner	22/185 (11.9)	0.53	(0.33 – 0.86)	0.56	(0.34 - 0.92)
Mean age at first sexual intercourse (years)		* 17.2	0.92	(0.85 – 0.99)		
Parity	0	195/988 (19.7)	1	-		
	≥ 1 child	10/105 (9.5)	0.43	(0.22 – 0.84)		
Mean age at first delivery (years)		* 21.8	1.01	(0.78 – 1.30)		
No. of lifetime partners	1	25/377 (6.3)	1	-	1	-
	2-4	118/558 (21.1)	3.78	(2.40 – 5.94)	4.15	(2.56 – 6.72)
	≥5	62/156 (39.7)	9.29	(5.54 – 15.57)	10.63	(6.16 – 18.36)
No. of partners in the last 6 months	0-1	181/1031 (17.6)	1	-		
	≥2	24/62 (38.7)	2.96	(1.73 – 5.07)		
Use of contraceptives in the last 6 months	No	35/261 (13.4)	1	-	1	-
	Yes	159/762 (20.9)	1.70	(1.14 – 2.53)	1.67	(1.09 – 2.54)
Use of condoms in the last 6 months	Always	68/346 (19.7)	1	-		
	Rarely	65/307 (21.2)	1.10	(0.75 – 1.60)		
	Never	61/367 (16.6)	0.81	(0.56 – 1.19)		
Previous Pap-test	No	135/671 (20.1)	1	-		
	Yes	70/420 (16.7)	0.79	(0.58 – 1.09)		

*mean.

**The following variables have been included in the multivariate model: marital status, living with, mean age at first sexual intercourse, mean age at first delivery, parity, number of lifetime partners, number of partners in the last 6 months, use of contraceptives in the last 6 months.

The genotyping was performed on 202/205 HR-HPV positive samples (three samples were not adequate because material was not sufficient for genotyping) and 194 (96%) showed positivity at least for one of the 18 types identifiable by the kit assay used for genotyping. In contrast, eight samples (4%) did not result positive at any of these 18 types: this proportion is in line with data reported in literature [17,18] and is mainly due to the cross-reactivity of the probe B with low-risk HPV types.

Among Hybrid Capture positive women HPV16 was the most prevalent type (30.9%), followed by 31 (19.6%), 66 (12.9%), 51 (11.3%), 18 (8.8%), 56 (8.8%) and 58 (8.3%); all the other types were detected at frequencies lower than 5.2% (Table 3); the cumulative rate of 16 and 18 was 38.7%.

**Table 3:** Distribution of HPV genotypes in HR-HC2 test positive women and overall population

**HPV genotypes**	**Number of infections**	**HR-HC2 test positive women (N = 194), %**	Overall population* (N = 1094), %
**16**	60	30.9	5.5
**31**	38	19.6	3.5
66**	25	12.9	2.3
**51**	22	11.3	2.0
**18**	17	8.8	1.6
**56**	17	8.8	1.6
**58**	16	8.3	1.5
**59**	10	5.2	0.9
**52**	9	4.6	0.8
**39**	9	4.6	0.8
**45**	8	4.1	0.7
53**	7	3.6	0.6
**33**	6	3.1	0.5
**68**	6	3.1	0.5
73**	3	1.5	0.3
**35**	2	1.0	0.2
82**	1	0.5	0.1
26**	0	0.0	0.0

* HPV type-specific prevalence has been extrapolated to overall population although it should be stressed that the genotyping was performed only on HR-HC2 positive samples.

** The prevalence of HPV 26,53,66,73,82 is probably underestimated because these HPV types are detected by the “Consensus High Risk HPV genotyping” assay, but they are not targeted by the HR-HC2 test.

The prevalence of HPV 26, 53, 66, 73, 82 is underestimated because these HPV types are detected by the “Consensus High Risk HPV genotyping” assay, but they are not targeted by HR-HC2 test; the detection of HPV 26, 53, 66, 73, 82 is due to an occasional cross-hybridization of HR-HC2 method or co-infection with HPV-targeted types, therefore they could more likely appear in multiple than single infections. For this reason we limited the analysis of multiple infections to the 13 HR-HPV types targeted by HR-HC2 test.

Out of 181 women infected by HR-HC2 targeted types, 37 (20.4%) had a multiple infection (36 infected by two types and one by three types). No difference in socio-demographic characteristics and sexual and reproductive history was found between women presenting a multiple and single infection. HPV16 was the most common genotype detected in multiple infections, followed by HPV31 and 18. Table 4 shows the distribution of HPV types in single and multiple infections; the proportion of single and co-infections by genotype is also reported: the proportion of infections occurring in conjunction with other types did not significantly differ by HPV type (Fisher’s exact test: p = 0.264).

**Table 4:** Distribution of HPV genotypes in single/multiple infections and proportion of single/multiple infections by type

HPV genotypes*	**Single infections**		**Multiple infections**	
	**n**	%**	**n**	%**
**16**	43	71.7	17	28.3
**31**	21	55.3	17	44.7
**51**	16	72.7	6	27.3
**18**	7	41.2	10	58.8
**56**	12	70.6	5	29.4
**58**	11	68.7	5	31.3
**59**	6	60.0	4	40.0
**52**	5	55.6	4	44.4
**39**	6	66.7	3	33.3
**45**	7	87.5	1	12.5
**33**	3	50.0	3	50.0
**68**	6	100.0	0	0.0
**35**	1	50.0	1	50.0
**All**	144	65.5	76	34.5

* All genotypes from single and multiple infections were computed individually.

** The denominator is represented by the total number of infections (single plus multiple) by genotype.

### Determinants of high risk HPV prevalence

The univariate analysis of socio-behavioural characteristics and HR-HPV infection is reported in Table 2. The risk of HR-HPV infection resulted significantly higher in women who declared a greater number of partners in lifetime (OR = 3.78, 95%CI: 2.40-5.94 if 2–4 partners and OR = 9.29, 95%CI: 5.54-15.57 if ≥5 partners) and in the last six months (OR = 2.96, 95%CI 1.73-5.07 if ≥2 partners), and in women who have used any contraceptive method in the last six months (OR = 1.70, 95%CI 1.14-2.53). In contrast, the risk of HR-HPV infection resulted significantly lower in women living with their partner compared to women living with parents/friends/alone (OR = 0.53, 95%CI: 0.33-0.86), in married women (OR = 0.30, 95%CI: 0.14-0.63) and women with children (OR = 0.43, 95%CI: 0.22-0.84). Also a higher age at the first intercourse showed a protective effect (OR = 0.92, 95%CI: 0.85-0.99).

The following variables were included in the multivariate model: marital status, “living with”, age at first sexual intercourse, age at first delivery, parity, number of lifetime partners, number of partners in the last six months, use of contraceptives in the last six months.

In the multivariate logistic regression model (Table 2), the effect of the number of lifetime sexual partner remained statistically significant (OR_adj_ = 4.15, 95%CI: 2.56-6.72 if 2–4 partners and OR_adj_ = 10.63, 95%CI: 6.16-18.36 if ≥5 partners). Also, living with the sexual partner and having used any contraceptive in the last six months remained significantly associated with the detection of HR-HPV infection (OR_adj_ = 0.56, 95%CI: 0.34-0.92 and OR_adj_ = 1.67, 95%CI: 1.09-2.54 respectively).

### Cytology

The pap-test result was available for 1,093/1,094 cervical smears tested for HR-HC2; 5.7% (62/1,093) of samples were inadequate, cytology was normal in 83.0% samples (907/1,093) and abnormal in 11.3% (124/1,093) samples: 62 ASCUS/AGUS (atypical squamous/glandular cervical cells of undetermined significance), 52 LSIL, 6 ASCH and 4 HSIL. The proportion of HR-HPV positivity was significantly higher in women with abnormal cytology (65/124, 52.4%) than in women with normal cytology (132/907, 14.6%) (p < 0,001). Among the 197 HPV-infected women with adequate cytology, 65 (33.0%) had an abnormal finding.

HPV16 was the most frequent virus strain in normal cytology, ASCUS/AGUS, LSIL and ASCH, detected in 29.3, 47.8, 24.2 and 66.7% of the samples respectively. All the four HSIL were HR-HPV positive and four different types were detected respectively (16, 18, 31, 33).

## Discussion

We found a prevalence of HR-HPV infections of 19% in a sample of Italian women aged 18–26 years. This percentage is in agreement with an Italian study which used a similar methodology to measure the HR-HPV prevalence in 18–24 year-old women from Tuscany Region [11]. This value is also included within the range (20-45%) reported in other national or worldwide studies for women younger than 25 years [12,19-22], although it is difficult to compare results of different studies because of different age groups and different procedures for enrolling participants. Not surprisingly, the rate of infection in this age group was higher than what was found in women aged 25–29 (14%) in New Technologies for CC screening (NTCC) randomized trials, which studied 45,000 women aged 25–60 years of northern and central Italy, participating in CC screening programmes [8,9]. This finding reflects a higher probability of acquiring new infections at younger ages, confirming the trend described in the international literature with a peak of HR-HPV prevalence in younger women and a continuous decline with increasing age [19-22].

Cervical cancer incidence is lower in the South than in the North of Italy, therefore a lower HR-HPV prevalence in the general population was expected in the South. Instead, we did not find any difference in HR-HPV prevalence rates between northern, central and southern regions of Italy, confirming what reported by Agarossi and coll. [22]. This finding should encourage the implementation, strengthening and promotion of cervical cancer screening programmes in southern Italy, where coverage and acceptance are still lower than in the rest of the country [23].

Genotype HPV16 was detected in 31% of HR-HC2 positive samples, followed by 31, 66, 51 and 18. This is well consistent with the results of a comprehensive meta-analysis on HPV positive women with normal cytology conducted by de Sanjosé and coll. [24], which reported HPV16, 31 and 18 among the five most common types worldwide. In Italy, HPV16 resulted to be the genotype most frequently detected in all studies and HPV 31 was frequently reported as the second most common genotype [11,22,25,26]. Regarding the other genotypes a high variability is reported among studies, which could be due to geographical differences, different target populations, different methods for genotyping and random fluctuation for quite rare genotypes [27,28].

According to the International Agency for Research on Cancer (IARC) classification, HPV16, 31, 51 and 18 are classified as “carcinogenic to humans”, while HPV 66 is classified as “possibly carcinogenic” [29]. It should be also mentioned that the prevalence of HPV 66, as of the other genotypes not targeted by HR-HC2 (HPV 53, 26, 73, 82), observed in the present study is plausibly underestimated because the detection of these types is only due to an occasional cross-hybridization of the method or co-infection with HPV-targeted types.

As expected, we found that women with cervical cytological abnormalities were at significantly increased risk for being infected by HR-HPV types than women with normal cytology (52 vs. 15%). Current Italian studies involving women with cytological abnormalities (different ages and enrolment criteria) reported HR-HPV prevalence of 34-68%, increasing with cytology severity: 24-56% in case of diagnosis of ASCUS/AGUS, 42-72% in LSIL and 73-95% in HSIL [21,22,26,27,30-32].

Co-infections of HR-HC2 targeted types represented the 20% of HPV positive samples. This value is included in the range (15-50%) reported in literature [33]; similar percentages of single and multiple HPV infections have been observed in young general populations [34,35]. It is still not clear whether co-infection with multiple types increases the risk of progression to cancer [22,36].

The multivariate analysis evidenced the role of the number of lifetime sexual partners as determinant of HPV infection, consistently with other studies [11,12,21,37], and strengthens the concept that that the most suitable age for HPV vaccination is the period preceding sexual activity. Living with partner had a protective effect against HR-HPV infection in the multivariate model; consistently with another Italian study [38], being married and having children showed a protective effect in the crude analysis, although not statistically significant in the multivariate analysis. All these variables could be considered markers of a steady relationship, explaining the association with a low HR-HPV prevalence. The use of any contraceptive method in the last six months remained associated to HR-HPV infection in the final model; we could suppose that women who used contraceptives in the last six months could have had a more intense sexual activity, with a higher risk of acquiring HPV, whose prevalence is higher in young ages. If considering only the use of condoms in the last six months, no association was detected between HR-HPV prevalence and this method of contraception; this point is debated and conclusions about the association between condoms’ use and HR-HPV prevalence are discordant among authors [11].

The major strengths of this study are that the sample was large and it targeted an age group that has not been investigated extensively.

Among the limitations, it should be mentioned the fact that our sample may not be entirely representative of Italy’s general population of females aged 18–26 years because only ten LHUs in six Regions participated, though local probabilistic samples were population-based and both urban and rural LHUs in northern, central and southern Italy were involved.

In addition the participation rate was 52%, therefore our findings could not be representative of the entire study population; however differences in socio-demographic characteristics between participants and people who declined participation were minimal.

The participation rate could be underestimated because we have excluded from the initial sample the 154 women who spontaneously declared not to be sexually active, but we do not know if other women, who declined participation, were virgin too (we did not collect this information). This age group represents a difficult target for prevention measures, because in Italy young adult women are not accustomed to being targeted by preventive programmes; moreover, enrolled women were offered the participation to a package of activities within the PreGio project, which is more difficult to be accepted than a single cervical smear. On the other hand, it should be noted that the study personnel had received special training and that a high number of attempts were made to contact non-respondents. Two other studies [11,12], which detected HPV prevalence of a sample of Italian women aged 18–24 years randomly selected from population registries, got a lower participation rate (15-22%). In these two studies letters of invitation were mailed to sampled women and a reminder was sent in case of no response, whereas we planned three phone calls and two home visits for non respondents.

As already mentioned, another limitation is that the prevalence of HPV 26, 53, 66, 73 and 82 could be heavily underestimated because they are not targeted by HR-HC2 test. In fact HPV 73 and 82 were observed only in co-infection with other HR-HPV types, suggesting that they could have been found only because coexisted with HPV types detected by HR-HC2 probes B (for this reason we excluded HR-HC2 not targeted types from the analysis of co-infections).

## Conclusion

We measured the genital HR-HPV prevalence in Italian women aged 18–26 years before the introduction of HPV vaccination across all the areas of Italy; we found a prevalence of 19%, higher than what detected for older women [19-22]. The use of the same molecular method and laboratory network of the NTCC studies [8,9], targeting women aged 25–60 years, allowed us to depict a comprehensive picture of HR-HPV prevalence in Italy from 18 to 60 years of age, observing in young adult women a peak of prevalence, which drops with increasing age. The findings support the choice of electing girls before the sexual debut as the primary target of HPV vaccination.

The HPV type distribution found in this study may represent a baseline picture; an accurate post-vaccine surveillance is, in fact, necessary to early detect a possible genotype replacement. The high prevalence of viral types other than vaccine-HPV types supports the necessity to guarantee the progression of CC screening programmes in vaccinated women. Studies linking screening programmes with vaccine immunization registries should be performed to evaluate the HPV vaccine effectiveness on vaccine-related and not-vaccine related types.

## Competing interests

The authors declare that they have no competing interests.

## Authors’ contributions

CG coordinated and monitored the project activities, analysed the data, interpreted the results, drafted and edited the manuscript. SDo designed the study, coordinated and monitored the project activities, interpreted the results and revised the manuscript. FC and GR participated in the study design, carried out the immunoassays, coordinated the activities of laboratories and revised the manuscript. SS designed the study, interpreted the results and revised the manuscript. SDe coordinated and monitored the project activities and revised the manuscript. MLCdA designed the study, coordinated and monitored the project activities in the first phase of the project and revised the manuscript. AB analysed the data and revised the manuscript. MPA, SB, NC, DF, AL, MCM, RN, EB participated in the study design and coordinated the activities in their local health units. EB, AG, VM, PP carried out the immunoassays. All authors read and approved the final manuscript.

## Pre-publication history

The pre-publication history for this paper can be accessed here:

http://www.biomedcentral.com/1471-2334/13/74/prepub
